# Phlebotomy in Ancient Times

**Published:** 1849-11

**Authors:** 


*Phlebotomy in ancient times.*—In the early ages some of the Abbeys had a bleeding house called *Phlebotomaria,* in which they had four general quarterly bleedings; and in the order of St. Victor, the brethren had five bleedings per annum. Half a century ago, bleeding was generally in fashion *spring* and *fall;* and surgeons were then never seen without a box of lancets and a red fillet. A fashionable phlebotomizing surgeon has been known to receive above a thousand guineas a year for this operation alone.—*Med. News.*

